# Elimination of schistosomiasis requires multifactorial diagnostics: evidence from high- and low-prevalence areas in the Nile Delta, Egypt

**DOI:** 10.1186/s40249-020-00648-9

**Published:** 2020-04-03

**Authors:** Hala Elmorshedy, Robert Bergquist, Amel Fayed, Wafaa Guirguis, Ensaf Abdel-Gawwad, Safaa Eissa, Rashida Barakat

**Affiliations:** 1 College of Medicine, Princess Nourah bint Abdulrahman University, P.O. Box: 84428, Riyadh, Postal Code: 11671 Saudi Arabia; 2 High Institute of Public Health, Alexandria University, Alexandria, Egypt; 3 Ingerod, SE-454 94 Brastad, Sweden

**Keywords:** Schistosomiasis, Transmission, Praziquantel, Knowledge attitude and practice, Utilization of health services, Poverty, Egypt

## Abstract

**Background:**

Schistosomiasis is one of the neglected tropical diseases (NTDs) selected for worldwide elimination in the near future. Egypt has made strong progress against its two endemic species of *Schistosoma mansoni* and *S. haematobium*. The former is prevalent in the Nile Delta with the latter dominating in the Nile south of Cairo. Innovative efforts are needed to reach the goal as further reduction of the prevalence has stalled due to ongoing transmission. In this study we aimed to explore the difference between low and high prevalence villages with regard to knowledge attitude and practice about schistosomiasis, utilization of health services, infection and transmission indices.

**Methods:**

A hybrid cross-sectional longitudinal study was conducted with three annual follow-ups conducted during 1994–1996. We used a representative systematic random sampling technique investigating 993 individuals from the high prevalence village and 614 from the low prevalence village. Data were analyzed using SPSS, comparing proportions with the Chi square test and means with the Student t test, and ANOVA.

**Results:**

Compliance of faecal sampling and chemotherapy was above 70% in both villages over the whole study period. Selective praziquantel treatment resulted in a significant reduction of prevalence and intensity of infection in both villages, dropping from 35.8% prevalence to 20.6%, in the low-prevalence village, and from 69.5 to 45.9% in the high-prevalence one. Intensity of infection at the base line was 30 eggs per gram (EPG) of stool in the low-prevalence village versus 105 EPG in the high-prevalence village. However, after the second round, reinfection rebounded by 22% in the high-prevalence village, while a slight improvement of the infection indices was demonstrated in the low-prevalence one. The level of knowledge was modest in both villages: people knew about self-protection and treatment, but not much about the role of human excreta for schistosomiasis transmission. While all participants maintained that using the water from the canals was inevitable, inhabitants in the high-prevalence village showed significantly lower scores reflecting higher water contact compared to the low-prevalence one. Many of them (67%) did not utilize the health centre at all compared to 26% of the people in the low-prevalence village. Interestingly, private clinics were seen as the primary source of health care by both villages, but more frequently so in the high-prevalence village (used by 87.2% of the inhabitants) compared to the low-prevalence one (59.8%).

**Conclusions:**

Even if chemotherapy works well as reflected by the observed downregulation of intensity of infection in both villages, reinfection continued due to difficulties to avoid water contact. Efforts must be made to make people understand the role of human excreta for transmission. There is also a need to make people better trust the medical services available.

## Background

The World Health Organization (WHO)'s roadmap for the global control of the neglected tropical diseases (NTDs) [1] encourages endemic countries to shift control activities of schistosomiasis towards elimination. With more than 800 million people in the world at risk and a third of them actually infected [2, 3], the prevalence of this disease is still alarmingly high in spite of larger amounts of the anthelmintic drug praziquantel (PZQ) distributed in the endemic areas than ever before [4]. The global burden of disease (GBD) study in 2010 [5] estimated 3.3 million disease-adjusted life years (DALYs) for schistosomiasis, while later updates [6] show sharply lower DALY scores down to 1.496 million in 2016. However, the true impact of schistosomiasis is much higher because of limitation of parasitological techniques to reveal light infection [7]. Indeed, molecular diagnostics indicate that the number of infections in an endemic area is considerably higher than that shown by egg-detection [8, 9].

Since the early 1980s the strategic approach to control schistosomiasis has been based almost exclusively on chemotherapy, often provided by mass drug administration (MDA), which has contributed to the decline of the DALY metric [6, 10]. However, despite the political support to maintain PZQ donations, genuine concerns have been raised regarding drug coverage as well as long-term sustainability [11, 12]. Although the excellent modality of PZQ that effectively controls disease morbidity [13–16], its negligent effect on disease transmission encumbers reaching the target of disease elimination. Increasing the dose and/or the number of doses and/or shifting to regular MDA cycles has not achieved interruption of transmission [10, 17, 18].

In the regional strategy for 2014–2020, issued by the WHO Regional Office for Africa (AFRO), it emphasizes the need for improved capacity of health systems, resource mobilization and financial sustainability of national NTD programmes in addition to enhancing monitoring, evaluation, surveillance and research [19]. Control of the aquatic snail intermediate host where and when transmission occurs should take precedence, and that means definition of water contact including acceptable management of sewage disposal, health education and improved water sanitation and hygiene (WASH) [20]. Indeed, several recent studies highlight the fact that knowledge and attitude about schistosomiasis remain insufficient and poor in many endemic areas [21–24]. Some people may assume that previous treatment prevents reinfection, which affects negatively water contact activity and compliance to repeated chemotherapy campaign In Zanzibar, as many as 75% of the children thought that there would be no risk of reinfection after treatment with PZQ [21]. Another study in northern Côte d’Ivoire and southern Mauritania, revealed that some people believed that schistosomiasis is caused by exposure to goat or dog urine in the environment, while others thought that the disease is transmitted by environmental elements such as sunshine and dirty water [25].

Adherence to MDA using PZQ is critical to the success of control strategies; overall, poor compliance is attributed to traditional, religious beliefs, poor acceptability of the drug, fear of side-effects and, above all, inadequate health education [26–29]. In the Philippines, PZQ coverage was only 43%, i.e. far below the WHO target of 75% [30]. Uncertainties on whether schistosomiasis can be treated, fear of praziquantel adverse reactions, misconceptions about alternative forms of treatment were all associated with higher odds of non-compliance [31]. Similarly, in Zanzibar, treatment compliance was only 50–60% [32].

Egypt has probably had the longest run of MDA with PZQ on the African continent, and it was claimed that that prevalence of *Schistosoma mansoni* in the Nile Delta has been reduced from 14.8% in 1993 to 2.7% in 2002 with a further decline to 1.5% in 2006 [33, 34]. However, this must have been an overestimation as the results represented pooled data and were carried out by stool examination using Kato-Katz standardized technique [35], which is known to miss light infections [9, 36]. Nevertheless, the Egyptian Ministry of Health and population (MoHP) upgraded the treatment strategy far beyond the WHO’s guidelines [14] aiming for interruption of transmission. However, transmission continued at an appreciable level in some foci [37, 38]. The MoHP is now planning to remap schistosomiasis in Egypt using a more sensitive diagnostic technique with the aim of identifying all areas of transmission where the elimination strategy should be applied. In 2018, a recent remapping survey using detection of the circulating cathodic antigen (CCA) from *S. mansoni* [39] was applied in the Nile Delta. This considerably more sensitive diagnostic tool detected *S. mansoni* infection in 31 districts out of 35 districts in the Nile Delta with prevalence ranging between 10 and 40% [40].

The role of primary health care is fundamental in sustaining a successful control strategy. Knowledge of the way people live in endemic communities and how they value and utilize the services provided by the primary health care facilities is crucial. At the 54th World Health Assembly (WHA54.19) in 2001, the planned minimum target for the year 2010 of PZQ coverage was given as 75% of all school-age children at risk, but the 65th Assembly meeting in 2012 (WHA65.21) noted that upon reaching that year, only 12.2% of people at risk benefitted from preventive chemotherapy [1]. Since then, however, the situation has improved and data for 2017 show that 44.9% of people requiring treatment for schistosomiasis globally were reached, while the proportion of school-aged children in this category amounted to 68% [41].

Few knowledge, attitude and practices (KAP) studies have been carried out in Egypt, and no studies looked at the peripheral health services. The main aim of the present study is to create community-based KAP portrays comparing a highly prevalent village to a low-prevalence community in the same region. In addition, we compared the healthcare service in the two villages and investigated the schistosomiasis transmission using multiple indicators not only limited to human infection, but also including malacological indices such as shedding and sentinel mice [42].

## Material and methods

### Study site and population

The data of the present study is part of the microlevel approach of Schistosomiasis Research Project (SRP) which was funded by the government of the USA and the MoHP aiming to improve control strategy of schistosomiasis [43]. Part of this project focused on the epidemiology of schistosomiasis using hybrid of cross-sectional prospective study designs, and included governorates in the Nile Delta, Nile Valley and new reclaimed areas [44].

Due to the the current, sometimes insecure situation, there was an unplanned, relatively long gap between the time of conducting the study and its finalization. However, this should not have caused any change of the level of transmission [40], the quality of peripheral healthcare services or the population education profile in the study area inrural Egypt.

**Fig. 1:**
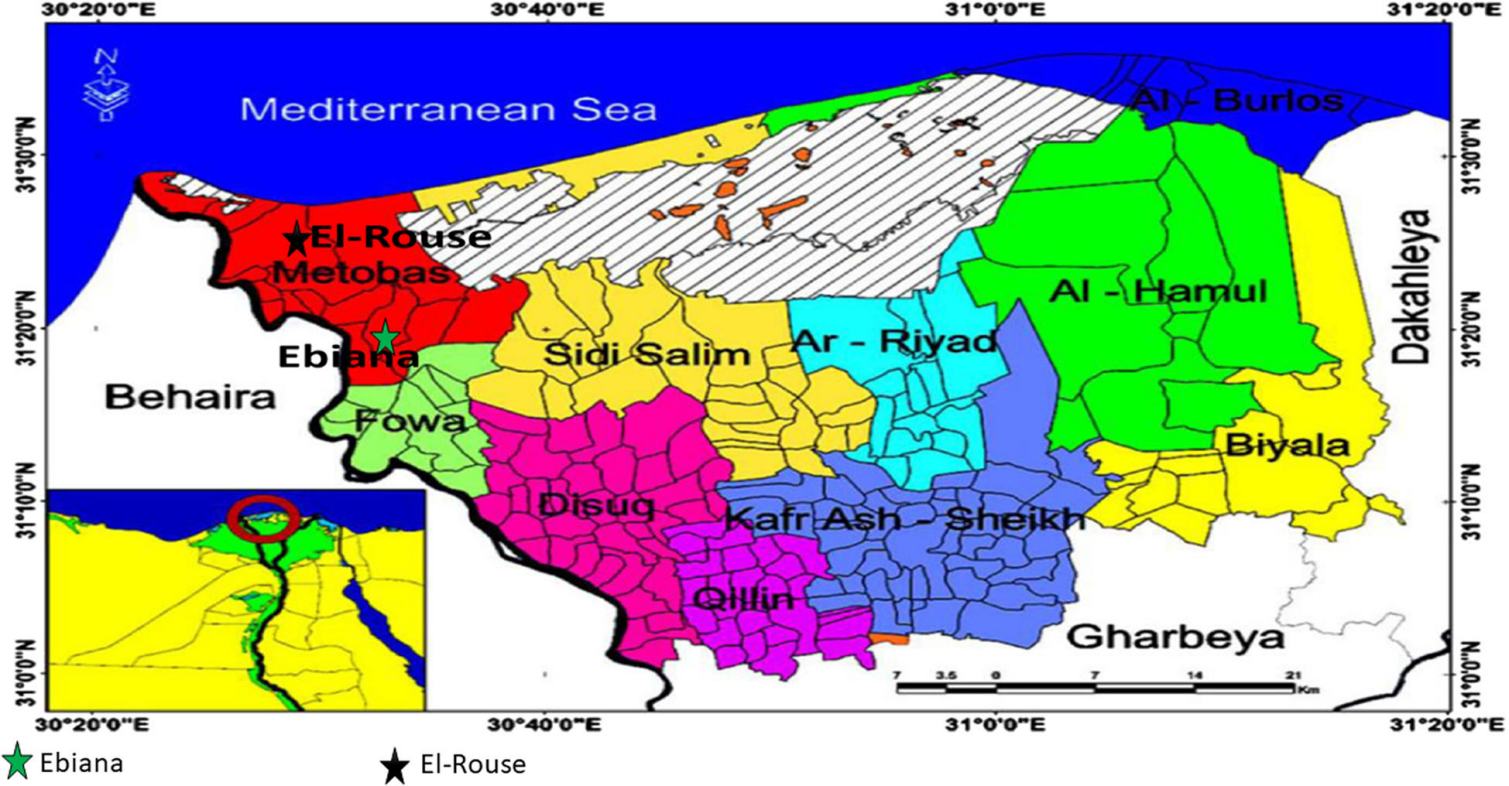
The District of Kafr El-Shiekh Governorate in Northern Nile Delta

This study was conducted in Kfer El-Sheikh Governorate, in the Northern part of the Nile Delta, Egypt (Fig. 1). Based on previous surveys in this region [45], two villages were selected: El-Rouse for high schistosomiasis prevalence and Ebiana for low.

El-Rouse is situated 1 km from the Rosetta branch of the River Nile and is a relatively new village built on reclaimed land. The village has only two schools; preparatory and mid-level. For water, the inhabitants depend on public taps in addition to canal water, but they lack a proper sanitary sewage disposal system. Most of the inhabitants are farmers producing mainly rice and vegetables. Ebiana is situated 20 km from El-Rouse and no more than 0.5 km from the Rosetta branch. This village is older and larger than El-Rouse and has a higher socioeconomic standard as well as an overall higher educational level. It has preparatory, mid- and high-level schools. There is a primitive sewage disposal system and most of the houses have access to clean water inside, however not always in adequate amounts, so some people depend partly on public tap water for domestic purposes. Farming is the main occupation also in this village and rice is the main crop. Each village has a peripheral health centre. From the health point of view, Ebiana is slightly better off than El-Rouse as it is just 1 km distant from Metobus City where there are multiple health care facilities including a governmental hospital and many private clinics.

### Study design

During the period from 1994 through 1996, We conducted a cross-sectional longitudinal study including individuals ≥ 6 years old. A stratified random sample, comprising 25% of houses in El-Rouse and 10% houses in Ebiana, was selected. In Ebiana, the sample included 993 individuals in 123 houses; in El Rouse, the sample included 614 individuals in 66 houses.

The sample size calculation was mentioned in the first report [43], published in 1998, showing that the sample size was selected by multistage stratified random sample, was calculated to detect a prevalence of *Schistosoma* sp. as low as 5% with an 80% precision and 90% confidence level. In Ebiana, the sample was 10% of the whole population and in Elrouse was 25%.The field examination included three main arms: KAP, parasitology and malacology.

### KAP study

The KAP questionnaire was designed by two specialists in health education and behavioural science, and the validity of the contents was confirmed by a panel of experts (Supp 1). Before the study, the questionnaire was piloted in the field to ensure face validity, while necessary modifications were implemented as necessary. Individuals ≥ 12 years old were interviewed by well-trained data collectors. Knowledge questions inquired about the disease, mode of transmission and preventive measures. This section included 13 questions with eight questions scored from 0 to 2 and 5 questions from 0 to 3. The maximum score was 31 with the total score levelled as: satisfactory (≥ 75% of the total), not quite satisfactory (74–50% of the total) and poor (< 50% of the total).

The attitude scale measured three domains including; perceived danger of canal water included seven items, perceived disease severity included three items, and perceived benefits of treatment included five items. In total, the scale comprised 15 items measured on a three point-Likert scale, i.e. agree; not certain; and don’t agree. The score ranged from 15 to 45, with higher scores indicating good attitudes. Water contact included six items scored from 1 to 3 (usually; sometimes; and never), The total score ranged from 6 to 18, where lower scores indicated more water contact. Experience of the study participants with regard to health services was investigated using questions that assessed the utilization of health services provided by the health center: four multiple option questions were designed to inquire about rating of the village health center as a source of care, place of latest stool analysis and treatment, and the principal source of health care. In addition, opinion of participants about the quality health services was investigated using 14 multiple options questions inquiring about physician’s performance, general proficiency of the health center, result of stool analysis, availability and cost of treatment, waiting and travel times.

### Parasitological study

The standard Kato-Katz methodology [35] was used for stool examination. Stool samples were collected annually at the end of transmission season and at the same time from the houses. Diagnosis of *S. mansoni* infection was based on examination of two consecutives stool samples from each person utilizing two slides containing 41.7 mg from each sample [46]. Egg counts from the four slides of the two consecutive samples were averaged and the egg per gram of stool (EPG) was computed. The outcome was used to calculate the geometric mean EPG of stool (GMEC). All EPG values were transformed into log_10_ + 1 to allow for zero counts, and the GMEC was computed as the anti-log_10_ of the mean of the log_10_ egg counts. This approach was used because intensity of infection does not follow the Poisson distribution, which makes the GMEC preferable compared to the arithmetic mean. PZQ-selective chemotherapy 40 mg/kg was offered by the team each year. The prevalence of infection was determined at the base line with follow-up prevalence, incidence and reinfection rates determined annually comparing two consecutive years. Incidence was computed as the percentage of positives among those who had tested negative in the previous year whilst reinfection was determined for those who tested positive despite being treated on the previous year [47].

### Malacological study

This part of the study focused on *Biomphalaria alexandrina*, the intermediate host of *S. mansoni.* Mapping of canals and drains in the two villages were performed to locate the transmission foci, i.e. hotspots, based on the density of infected snails and water contact activities. Only one snail survey per year was conducted in each village during the transmission season (May–December) at selected stations along a total length of 16.8 km of water courses in each village. Snails were collected with scoops using three dips from each station. At the central lab, the snails were identified with respect to species and examined for Schistosome Cercariae using the shedding technique [48]. In addition, sentinel mice experiments was conducted where a group of 10 white albino mice weighing 17–25 g were kept in water contact at the selected stations using floating cages with immersed bottoms for two hours between 10 AM and 2 PM. The mice were perfused eight weeks after exposure to determine the risk of infection at each station.

### Statitical analysis

Data were analyzed using SPSS, version 21.0 (IBM, New York, NY, USA). Qualitative variables were described in percentages, while quantitative variables were described in means ± *SD*. Intensity of infection was expressed as GMEC. We used Pearson’s Chi square test to compare between proportions, and the independent student *t*-test or ANOVA as appropriate to test for difference between quantitative variables. All tests were two-sided, and *P* <  0.05 was considered statistically significant.

## Results

The prevalence of *S. mansoni* was 35.8% at the baseline in Ebiana and nearly double that (69.5%) in El-Rouse. Additionally, the intensity of infection in El-Rouse was almost triple that found in Ebiana. Compliance was above 70% in both villages over the whole study period (Table 1).

**Table 1:** Sample size, compliance of stool samples and *Schistosoma mansoni* infection indices in high and low prevalence villages, Nile Delta Egypt

Variable	Low prevalence village (Ebiana)	High prevalence village (El-Rouse)
Households examined	123	66
Population size examined	993	614
**Parasitological data at baseline** **(Round I)**		
Compliance of stool samples%	85.9	81.1
Prevalence%	35.8	69.5
GMEC/EPG of infected people	30.1	104.7
**Parasitological data of First annual Follow-up (Round II)**		
Compliance of stool samples%	73	70
Prevalence%	20.6	45.9
GMEC/ EPG of infected people	20	41.7
Incidence%	10.5	21.4
Reinfection%	35.1	58
**Parasitological data of second annual Follow-up (Round III)**		
Compliance of stool samples%	70	72
Prevalence%	15.3	48.8
GMEC/ EPG of infected people	20.9	41.7
Incidence%	9.2	28.5
Reinfection%	32.9	70.6

*GMEC* Geometric mean egg count, *EPG* Egg per gram of stool

Annual application of PZQ treatment for cases testing positive resulted in a significant reduction of prevalence and intensity of infection in both villages after the first intervention. However, while the prevalence fell faster in Ebiana than in El-Rouse, and continued to decrease there over the whole study period, the prevalence in the latter actually rebounded after the second round (Table 1). The force of transmission, as measured by incidence and reinfection, demonstrated a slight improvement in Ebiana, whilst reinfection increased from 58 to 70.6% and incidence shoot-up up from 21.4 to 28.4% in El-Rouse (Table 1). However, the intensity of infection remained very similar in both villages in the follow-up years after falling compared to the baseline, particularly in El-Rouse.

**Fig. 2:**
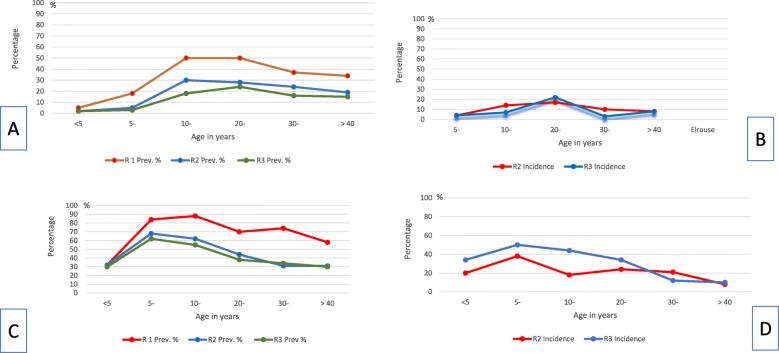
Parasitological data of the two villages according to age at baseline, and during first and second annual follow-ups (**a**) Prevalence in Ebiana Village; (**b**) Incidence in Ebiana Village; (**c**) Prevalence in EI-Rouse; (**d**) Incidence in EI-Roise Village. Prev.: Prevalence

The trend of prevalence and incidence according to the participants’ ages shows that the prevalence peaked at the school-age level in both villages. Although no infection was detected among children younger than five years in Ebiana, ominously, more than quarter of the children of the same age were infected in El-Rouse. Incidence and reinfection data reveal that children below five were negative at the first annual follow-up in Ebiana, while more than one third of those of the same age category were re-infected, and one fifth were newly infected, in El-Rouse. Following PZQ chemotherapy, the first annual follow-up revealed an initial improvement followed by either stationary results, as shown in Ebiana, or deterioration, as shown in El-Rouse. This trend was similar among all age groups (Fig. 2).

The malacological and sentinel mice data are shown in Table 2. The number of transmission foci in El-Rouse exceeded that of Ebiana, i.e. in eight out of 14 stations, *B. alexandrina* snails tested positive for cercarial shedding in the former village compared to four out of 10 stations in the latter. Five of the eight infected test sites were drains in El-Rouse versus only one out of the four in Ebiana. The distribution of infected mice coincided with the foci harbouring infected snails. Furthermore, a longer canal area was infected in El-Rouse compared to Ebiana, where the transmission sites were closer to each other.

**Table 2:** Distribution of snails and sentinel mice in the two villages

Ebiana Village (low prevalence)		
Site No. & type	Frequency & (%) of infected snails	Frequency & % of infected mice
1-C^a^	5.0 (2.9)	0.0 (0)
2-C	4.0 (5.5)	6.0 (20.6)
3-C	0.0 (0.0)	0.0 (0.0)
4-C	0.0 (0.0)	0.0 (0.0)
5-D^b^	2.0 (1.4)	6.0 (53.8)
6-D	0.0 (0.0)	0.0 (0.0)
7-C	0.0 (0.0)	0.0 (0.0)
8-C	0.0 (0.0)	0.0 (0.0)
9-C	2.0 (4.7)	2.0 (10.0)
10-C	0.0 (0.0)	0.0 (0.0)
**El-Rouse Village (high prevalence)**		
1-C	1.0 (6.0)	4.0 (6.8)
2-D	0.0 (0.0)	0.0 (0.0)
3-D	1.0 (0.15)	0.0 (0.0)
4-C	0.0 (0.0)	0.0 (0.0)
5-C	0.0 (0.0)	1.0 (2.0)
6-C	0.0 (0.0)	3.0 (12.0)
7-C	0.0 (0.0)	1.0 (3.6)
8-C	3.0 (6.3)	5.0 (26.4)
9-C	2.0 (1.7)	5.0 (10.0)
10-D	2.0 (0.99)	0.0 (0.0)
11-D	4.0 (0.12)	2.0 (7.8)
12-C	0.0 (0.0)	2.0 (12.3)
13-C	2.0 (0.32)	1.0 (1.7)
14-D	1.0 (0.5)	1.0 (3.4)

^a^C: Canals

^b^D: drains

Results demonstrate different levels of general knowledge on schistosomiasis between the two villages. While both villages showed modest levels of knowledge (Ebiana: 16.17 ± 7.75 and El-Rouse: 12.83 ± 7.69) out of the maximum score of 31, the participants from Ebiana answered almost all questions referring to knowledge assessment more satisfactorily. Focusing on knowledge related to the individual, ways of self-protection and treatment were the most frequently correctly answered questions. Nevertheless, the role of human excreta and mode of transmission were the least correctly understood by all villagers (Table 3).

**Table 3:** Level of knowledge about schistosomiasis in the two villages

Knowledge Question	Knowledge level (Percentage)					
	Poor		Unsatisfactory		Satisfactory	
	Ebiana	Elrouse	Ebiana	Elrouse	Ebiana	Elrouse
What is bilharziasis?	27.7	46.0	15.6	9.6	56.7	44.4
Which organs are severely affected?	8.6	13.0	21.0	26.6	60.4	60.4
How do people get infected?	5.3	3.5	41.6	23.9	53.1	72.6
How does bilharzia infect via canal water?	17.4	10.6	48.2	63.0	34.4	26.4
Which sources pollute the canal water?	36.6	29.0	3.5	8.8	59.6	61.2
How does human excreta reach the canal?	64.0	79.5	20.5	14.4	15.5	6.1
What, and where are snail habitat?	40.5	55.1	1.8	0.2	57.7	44.7
What is the role of snails in the life cycle?	58.9	68.9	2.8	2.6	38.3	28.5
How can one identify bilharziasis?	21.8	33.8	31.3	28.5	47.0	37.7
What are the types of available treatment	0.3	1.9	0.5	0.0	99.0	98.1
How can one be sure of cure after being treated?	21.9	38.6	11.6	23.7	66.5	37.9
How can you protect water stream from Bilharzia?	33.6	41.4	9.9	9.1	56.5	49.5
How can you protect yourself From Bilharzia?	7.2	14.4	3.1	7.2	89.6	78.4
Overall Knowledge	43.8	59.2	35.5	30.4	20.7	10.4

The total maximum score equaled 31: satisfactory (≥ 75%), Unsatisfactory (74–50%) and poor (< 50%)

The total knowledge score did not only significantly differ between the two villages, but varied also significantly according to age, gender, educational background and occupation. Poor knowledge was more prevalent among older age, females, lower educational levels, farmers and non-working groups (Table 4).

**Table 4:** Mean knowledge scores according to personal factors in the two villages

Variable	Ebiana *N* = 639	El-Rouse *N* = 377
	Mean ± *SD*	Mean ± *SD*
**Age group**		
12–19	16.37 ± 7.09	12.99 ± 7.69
20–29	18.65 ± 7.23	15.12 ± 7.63
30–39	16.14 ± 7.95	12.31 ± 7.39
40–49	15.07 ± 7.74	11.30 ± 7.27
50+	12.77 ± 7.00	9.63 ± 7.15
Statistics: *F* & (*P* value)	9.80 (< 0.001)	6.19 (< 0.001)
**Gender**		
Male	17.3 ± 7.75	16.06 ± 7.47
Female	15.15 ± 7.62	10.12 ± 6.79
Statistics: *t* & (*P* value)	12.59 (< 0.001)	64.77 (< 0.001)
**Education**		
Illiterate/read & write	13.33 ± 6.89	10.96 ± 6.82
Primary	15.66 ± 7.70	18.41 ± 7.01
Preparatory	21.21 ± 6.49	22.10 ± 4.94
Secondary	21.74 ± 5.4	21.44 ± 5.06
University	25.56 ± 4.29	26.33 ± 3.22
Statistics: *F* & (*P* value)	57.94 (< 0.001)	31.69 (< 0.001)
**Occupation**		
House wife	13.36 ± 7.06	9.5 ± 6.42
Fishermen	15.00 ± 0.0	15.18 ± 6.91
Farmers	15.59 ± 6.19	13.66 ± 6.95
Not working	15.83 ± 7.69	10.12 ± 7.09
Drivers	16.22 ± 7.10	15.92 ± 8.71
Students	18.8 ± 7.68	20.11 ± 6.43
Professionals	23.12 ± 6.19	22.60 ± 4.22
Others	15.36 ± 7.75	15.10 ± 7.14
Statistics: F & (*P* value)	13.70 (< 0.001)	16.27 (< 0.001)
**Total knowledge score (mean ±** ***SD*)**	**16.17 ± 7.75**	**12.83 ± 7.69**
**Statistics: students*t*test, (*P***-value**)**	**8.73 (< 0.001)**	

*SD* Standard deviation

The mean attitude scores regarding prevention and control of schistosomiasis as perceived by Ebiana and E-Rouse inhabitants is displayed in Table 5. Despite the favourable attitude reported by both villagers across all domains, the participants maintained that they could not avoid using canals even if public water taps and indoor water are available. Many reasons were reported, such as no water in the tap water for days on end, poor quality of tap water when available, crowding around public taps in addition to exposure to canal water as a natural occupational part of various activities. Ebiana’s inhabitants perceived the danger of water contact more profoundly than those in El-Rouse while the inhabitants of the latter village perceived the benefit and importance of treatment more positively than those living in Ebiana.

**Table 5:** Mean attitude score regarding schistosomiasis in the two villages

Attitude Scale	Ebiana *N* = 639	El-Rouse *N* = 377	*t* value
**Perceived danger of using canals’ water**			
Despite the presence of public tap water, people still use canals	2.73	1.88	16.26*
Despite having clean water indoors, people still use canals	2.28	2.26	11.26*
People claim that they are used to handle canal’s water, and they had never been infected	2.55	2.57	0.39
Belief that just washing cloths and utensils in canals, doesn’t cause bilharziasis	2.84	2.92	2.80*
Belief that irrigating land bare-footed doesn’t cause bilharziasis	2.83	2.95	4.55*
Belief that swimming in canals once/twice doesn’t cause bilharziasis	2.87	2.95	3.23*
Belief that just washing hands, legs or ablution in canals doesn’t cause bilharziasis	2.27	2.85	3.43*
**Subtotal score (out of 21)**	**19.33**	**18.39**	**5.51**
**Perceived severity of bilharziasis**			
Belief that bilharziasis is not a serious infection with potentially grave symptoms	2.99	2.98	0.99
Belief that bilharziasis should not make you seriously worried	2.84	2.92	2.48*
Belief that bilharziasis does not seriously affect person’s work capacity	2.95	2.96	0.52
**Subtotal score (out of 9)**	**8.78**	**8.86**	**1.87**
**Perceived benefits of treatment and avoiding canals’ water**			
Belief that you would never be infected if you had never used canal water	2.03	2.59	9.22
Belief that avoiding urination and defecation in streams will prevent bilharziasis	2.81	2.88	1.95
Belief that medicine from the Health center can cure bilharziasis	3.00	2.96	3.49*
Belief that that repeated urine and stool examination is important	2.97	2.99	1.54
Belief that there is no need for treatment because you are going to be infected again	2.97	2.99	1.54
**Subtotal score (out of 15)**	**13.79**	**14.41**	7.97*
**Overall mean total score ±** ***SD*(out of 45)**	41.89 ± 3.43	41.60 ± 2.83	1.1

*P < 0.005

*SD* Standard deviation

As for water contact practices, Ebiana’s inhabitants showed significantly higher scores reflecting less water contact compared to El-Rouse villagers. However, people seemed to use the canals for many different domestic and occupational purposes, i.e. to survive, farm, fish and wash (Table 6).

**Table 6:** Mean score of water contact practices

Water contact practices^c^	Ebiana		El-Rouse		*t* value
	No.	Mean ± *SD*	No.	Mean ± *SD*	
Farming	814	2.59 ± 0.75	485	2.31 ± 0.86	5.89^d^
Ablution/hand washing	814	2.63 ± 0.69	485	2.41 ± 0.78	5.29^d^
Swimming	814	2.81 ± 0.50	485	2.55 ± 0.75	7.41^d^
Washing cloths^a^	424	2.80 ± 0.47	255	1.60 ± 0.85	23.69^d^
Washing animals^b^	390	2.55 ± 0.64	230	2.32 ± 0.77	4.01^d^
Bringing water from canals	814	2.76 ± 0.54	485	1.70 ± 0.78	28.06^d^
Fishing	814	2.82 ± 0.44	485	2.78 ± 0.52	1.48

^a^Asked to females only

^b^Asked to males only

^c^Lower scores indicate more water contact

^d^*P* < 0.005 *SD*: Standard deviation

Findings related to source of health care indicated clearly that the role of the health centre differed substantially between the two villages. In Ebiana, the village health centre was found to be the primary or secondary source of care more frequently (34.6 and 37.9%, respectively), while the corresponding figures were 7.6 and 23.5%, respectively, in El-Rouse. As many as 66.9% of the inhabitants in El-Rouse, compared to 26.3% in Ebiana, said that they never used the health centre. Private clinics were found to be the primary source of health care by the greater proportion at both villages, but more frequently at El-Rouse (87.2%) than at Ebiana (59.8%). Concerning analysis and treatment for schistosomiasis, it appears that the current project was the site of the last analysis and treatment for the greater proportion at Ebiana (78.5 and 54.8%, respectively) as well as El-Rouse (76.9 and 64.4% respectively) (Table 7).

**Table 7:** Health services utilization by Ebiana and El-Rouse villagers

	Ebiana *N* = 818	El-Rouse *N* = 486
**Rating of health center as a source of health care**		
Primary source	283 (34.6)	37 (7.6)
Secondary source	310 (37.9)	114 (23.5)
Not a source	215 (26.3)	325 (66.9)
Never sought health care	10 (1.2)	10 (2.1)
**Primary source of health care**		
Private clinic	489 (59.8)	424 (87.2)
Health center	283 (34.6)	37 (7.6)
MOPH hospitals	24 (2.9)	13 (2.7)
Health insurance unit	11(1.4)	1 (0.2)
Other sources	11(1.3)	11(2.3)
**Place of last stool analysis**	***N*** **= 804**	***N*** **= 463**
Project	631(78.5)	356 (76.9)
Health center	110(13.7)	42(9.1)
Private physician	17(2.1)	44(9.5)
MOPH hospital	19(2.4)	8(1.7)
Private laboratory	15(1.9)	4(0.9)
School	6(0.7)	1(0.2)
Other governmental facility	6(0.7)	8(1.7)
**Source of last treatment**	***N*** **= 516**	***N*** **= 388**
Project	283 (54.8)	250 (64.4)
Health center	119 (23.0)	34 (8.8)
Private physician	9 (1.8)	22 (5.7)
MOPH hospital	35 (6.8)	27 (7.0)
Pharmacy	66 (12.8)	48 (12.3)
School	0(0.0)	1 (0.3)
Other governmental facility	4 (0.8)	6 (1.5)

Findings related to the opinion of respondents towards several aspects of the services available at the village health centres indicate a more favourable score at the lower-prevalence village Ebiana, which has a greater utilization of the health centre than El-Rouse. The difference was particularly marked in relation to opinion with respect to the physicians. Thus, 72.6–85% at Ebiana thought that the physicians there examine the patients professionally and treat them well in general as well as spend enough time with the patients, explains conditions and medications and are generally competent, while the corresponding figures ranged between 30.8 to 41.6% in El-Rouse (Table 8).

**Table 8:** Opinion of respondents about health services provided by the village health center

	Ebiana *N* = 643	El-Rouse *N* = 377
**General handling of the patient by the physician**		
Good	551 (85.7)	143 (37.9)
Average	48 (7.5)	35 (9.3)
Bad	28 (4.4)	167 (44.3)
Don’t know	16 (2.5)	32 (8.5)
**Subjected to physical examination**		
Yes	475 (73.9)	133 (35.3)
Sometimes	51 (7.9)	48 (12.7)
No	89 (13.8)	140 (37.1)
Don’t know	28 (4.4)	56 (14.9)
**Time spent with patient by the physician**		
Enough	467 (72.6)	116 (30.8)
Sometimes enough	15 (2.3)	33 (8.8)
Not enough	109 (17.0)	160 (42.4)
Don’t know	52 (8.1)	68 (18.0)
**Patients given chance to explain condition and symptoms**		
Yes	543 (84.4)	139 (36.9)
Sometimes	20 (3.1)	43 (11.4)
No	50 (7.8)	126 (33.4)
Don’t know	30 (4.7)	69 (18.3)
**Condition and medications prescribed explained by the physician**		
Yes	530 (82.4)	129 (34.2)
Sometimes	26 (4.0)	41 (10.9)
No	60 (9.3)	137 (36.3)
Don’t know	27 (4.2)	70 (18.6)
**General proficiency of the health center**		
Competent	518 (80.6)	157 (41.6)
Average	92 (14.3)	162 (43.0)
Don’t know	33 (5.1)	58 (15.4)
**Treatment in general**		
Good	508 (79.0)	143 (37.9)
Average	53 (8.2)	35 (9.3)
makes me not want to seek treatment at health center	61 (9.5)	167 (44.3)
Don’t know	21 (3.3)	32 (8.5)
**Results of analysis**		
Mostly right	403 (63.5)	189 (50.3)
Mostly wrong	94 (14.8)	85 (22.5)
Sometimes wrong	13 (2.1)	3 (0.8)
wrong if extra fees not paid	31 (4.9)	13 (3.4)
Don’t know	93(14.7)	87 (23.0)
**Bilharzia tablets available**		
Yes	382 (60.2)	158 (41.9)
Sometimes	124 (19.6)	85 (23.7)
No	48 (7.6)	37 (10.3)
Don’t know	80 (12.6)	86 (24.0)
**Cost of diagnosis and treatment of Bilharziasis**		
Reasonable	568 (89.6)	278 (73.7)
A lot	13 (2.1)	34 (8.9)
Don’t know	53 (8.4)	65 (17.3)
**Accessibility (among users of centers only)**	***N*** **= 439**	***N*** **= 116**
**Travel time to the health center**		
1–5 min	231 (52.6)	17 (14.7)
6–10 min	125 (28.5)	21 (18.1)
11–15 min	62 (14.1)	44 (37.9)
16+ minutes	21 (4.8)	34 (29.3)
**Opinion about travel time to the health center**		
Reasonable	433 (98.6)	92 (79.3)
Too much	6 (1.4)	24 (20.7)
**Waiting time before seeing the physician at latest visit**		
1–15 min	332 (75.6)	39 (33.6)
16–30 min	56 (12.7)	22 (19.0)
31–60 min	31 (7.1)	19 (16.4)
61+ minutes	11 (2.5)	28 (24.1)
Doctor did not arrive	9 (2.1)	8 (6.9)
**Opinion about waiting time at latest visit**		
Reasonable	433 (98.6)	91 (78.4)
Too much	6 (1.4)	25 (21.6)

The health centres at both villages were felt to be reasonably accessible to their users, but the centre at Ebiana more so than in the neighbouring village. The travelling time was only 1–5 min for 52.6% of the users at Ebiana compared to 14.7% at El-Rouse, and it was rated reasonable by 98.6 and 79.3%, respectively. Waiting time in the centre to see a physician during the latest visit was rated as reasonable by 98.6% compared to 78.4% at Ebiana and El-Rouse, respectively. Waiting time did not exceed 15 min for 75% of the users at Ebiana versus 33.6% at El-Rouse, while it exceeded 1 hour at Ebiana for 2.5% compared to 24.1% at El-Rouse.

## Discussion

Primary care healthcare centres maintain the NTD control strategies in the field on a day-to-day basis dispensing health education, diagnosis and treatment under the supervision of the MoHP. In order to visualize how this role functions in the periphery with regard to schistosomiasis, this is the first Egyptian study investigating the influence of knowledge and practises on the infection status over a 3-year period in two villages in Egypt’s Nile Delta, one of the remaining areas in the country endemic for *S. mansoni*. Despite the generally favourable attitude towards the health centres and their work reported by villagers across all domains, the participants felt that they could not completely avoid canal water contact. Although most villagers participating in the study had an understanding of the type of infections lurking in the canal water, they downplayed the risk. Local knowledge how schistosomiasis is transmitted and prevented and view of the healthcare services available are crucial to achieve the target of effective control. In accordance with our results, previous studies highlight the insufficient knowledge about schistosomiasis in the endemic areas [21, 22, 25]. Although people responding to the questions in the questionnaire had a good general understanding of the disease, most of them still lacked knowledge of specifics, such as the parasite’s life cycle and the high risk even of the slightest water contact. This leads to a false feeling of safety indicating that education needs to be strengthened. In addition, tap water provision must be improved encouraging people to abstain from other forms of water contact. This was underlined by the more favourable scores obtained at the low-prevalence village and its greater utilization of the health centre and more positive attitude towards the physicians there. Access and waiting time, which were more favourable at the low-prevalence village, might also play a role.

Lack of knowledge is the ultimate outcome of poverty and the attenuated role of the healthcare services. They both perpetuate the risk of reinfection and negatively affect treatment seeking. This clearly applies to sub-Saharan Africa, having the highest prevalence of schistosomiasis coupled with the lowest income per-capita [49], and to the situation in Yemen [38]. This is in contrast to developed countries which succeeded in eliminating this infection; for example, Japan [50] and modern-day China [51], which has made a remarkable progress towards elimination. Also, in the Middle East and North Africa (MENA) region, the economic transition in Saudi Arabia played an immense role in diseases elimination [38].

The malacological study, based both on cercarial shedding and sentinel mice showed the whereabouts of the hotspots and the level of transmission experienced in the two villages. The number of foci in the high-prevalence village exceeded that of the low-prevalence one, and many more places tested positive for cercarial shedding in the low-prevalence village compared the high-prevalence one, which was also reflected by the distribution of mice infection. In El-Rouse, most of the infected snail stations were drains, an observation clearly reflecting that this village lacks sewerage system and human excreta are just poured into drains — the same as in communities where poverty is rampant. Therefore, despite PZQ treatment for two successive rounds, infection indices shoot-up in the second round after the initial downregulation achieved in the first round. In contrast, the downward trend in infection indices in Ebiana continued till the end of the study. Therefore, despite the complex interrelation between the burden of schistosomiasis as one of the neglected tropical diseases and poverty, the disease could be eliminated simply by improved sanitation [49]. It is worth mentioning that both Ebiana and El-Rouse share the same geographic situation — being located at the end of the stream of the Rosetta branch of the Nile River, they both have the same farming practices where rice is the predominant crop, yet the two villages differ with respect to sewerage system and water supply as well as appreciation of health services.

### Limitations of the study

Diagnosis of schistosomiasis was based on parasitological techniques using two consecutive samples instead of the ideal three consecutive samples. Thus, the infection indices are likely to be underestimated. Because rural communities in the Nile Delta share similar characteristics thus, we believe that our findings are generalizable to the entire rural population. However, further studies are required to update the available knowledge.

## Conclusions

Application of PZQ chemotherapy resulted in a significant reduction of prevalence and intensity of infection in both villages but rebounded after the second round in the high-prevalence village. Nevertheless, transmission continues at an appreciable level in both villages. KAP data revealed an overall modest level of knowledge in both villages, but population knowledge was poor with regard to disease transmission, and the role of human excreta in contaminating water bodies. The high-prevalence village showed more water contact activities compared to the low-prevalence village. People in the high-prevalence village were considerably more negative about the health services offered than in the low prevalence village.

## Supplementary information

**Additional file 1.** Data collection sheet.

## Data Availability

The datasets used and/or analyzed during the current study are available from the corresponding author on reasonable request.

## Abbreviations

AFRO: WHO Regional Office for Africa
CCA: Circulating cathodic antigen
DALYs: Disease-adjusted life years
EPG: Egg Per Gram
GBD: Global burden of disease
GMEC: Geometric Mean Egg per gram
KAP: Knowledge, attitude and practices
MDA: Mass drug administration
MENA: Middle East and North Africa
MoHP: Ministry of Health and Population
NTDs: Neglected tropical diseases
*OR*: Odds Ratio
POC-CCA: Point of care test based on the schistosome cathodic circulating antigen
PZQ: Praziquantel
SRP: Schistosomiasis Research Project
USA: United States of America
USAID: United State of America International Development
WASH: Water Sanitation and Hygiene
WHA: World Health Assembly
WHO: World Health Organization

## References

[1] WHO. Accelerating work to overcome the global impact of neglected tropical diseases. A roadmap for implementation. https://www.who.int/neglected_diseases/NTD_RoadMap_2012_Fullversion.pdf Accessed 22 Feb 2020.

[2] Colley DG, Bustinduy AL, Secor WE, King CH (2014). Human schistosomiasis. Lancet (London, England).

[3] Steinmann P, Keiser J, Bos R, Tanner M, Utzinger J (2006). Schistosomiasis and water resources development: systematic review, meta-analysis, and estimates of people at risk. Lancet Infect Dis.

[4] WHO. Schistosomiasis.https://www.who.int/news-room/fact-sheets/detail/schistosomiasis.Accessed 10 Feb 20195.

[5] Murray CJ, Vos T, Lozano R, Naghavi M, Flaxman AD, Michaud C (2012). Disability-adjusted life years (DALYs) for 291 diseases and injuries in 21 regions, 1990-2010: a systematic analysis for the global burden of disease study 2010. Lancet.

[6] GBD 2016 DALYs and HALE Collaborators (2017). Global, regional, and national disability-adjusted life-years (DALYs) for 333 diseases and injuries and healthy life expectancy (HALE) for 195 countries and territories, 1990–2016: a systematic analysis for the Global Burden of Disease Study 2016. Lancet.

[7] King CH, Galvani AP (2018). Underestimation of the global burden of schistosomiasis. Lancet.

[8] Ortu GNO, Clements M, Kayugi D, Campbell CH, Lamine MS, Zivieri A, et al. Countrywide Reassessment of *Schistosoma mansoni* Infection in Burundi Using a Urine-Circulating Cathodic Antigen Rapid Test: Informing the National Control Program. Am J Trop Med Hyg. 2017. 10.4269/ajtmh.4216-0671. 10.4269/ajtmh.16-0671 PMC5361543 28115675

[9] Colley DGBS, Campbell C, King CH, Tchuem Tchuenté LA, N'Goran EK, et al. A five-country evaluation of a point-of-care circulating cathodic antigen urine assay for the prevalence of *Schistosoma mansoni*. Am J Trop Med Hyg. 2013. 10.4269/ajtmh.4212-0639.. 10.4269/ajtmh.12-0639 PMC3592520 23339198

[10] Secor WE, Colley DG. When Should the Emphasis on Schistosomiasis Control Move to Elimination? Trop Med Infect Dis. 2018;3(3). 10.3390/tropicalmed3030085. 10.3390/tropicalmed3030085 PMC6161309 30274481

[11] Parker M, Allen T (2014). De-politicizing parasites: reflections on attempts to control the control of neglected tropical diseases. Med Anthropol.

[12] Fenwick A, Savioli L (2011). Schistosomiasis elimination. Lancet Infect Dis.

[13] WHO/Department of control of neglected tropical diseases. Schistosomiasis and soil-transmitted helminthiases: number of people treated in 2016. https://www.who.int/neglected_diseases/resources/who_wer9249/en/.Accessed 22 Feb 2020.

[14] Barakat RM (2013). Epidemiology of Schistosiasioms in Egypt: travel through time: review. J Adv Res.

[15] Muhumuza S, Kitimbo G, Oryema-Lalobo M, Nuwaha F (2009). Association between socio economic status and schistosomiasis infection in Jinja District, Uganda. Tropical Med Int Health.

[16] Utzinger J, Zhou XN, Chen MG, Bergquist R (2005). Conquering schistosomiasis in China: the long march. Acta Trop.

[17] Bergquist R, Elmorshedy H. Artemether and Praziquantel: origin, mode of action, impact, and suggested application for effective control of human Schistosomiasis. Trop Med Infect Dis. 2018. 10.3390/tropicalmed3040125. 10.3390/tropicalmed3040125 PMC6306701 30572592

[18] Tchuem Tchuente LA, Rollinson D, Stothard JR, Molyneux D (2017). Moving from control to elimination of schistosomiasis in sub-Saharan Africa: time to change and adapt strategies. Infect Dis Poverty.

[19] AFRO**,** 2013**.** Towards an African Region free from Neglected Tropical Diseases**.** https://afro.who.int/news/towards-african-region-free-neglected-tropical-diseases. Accessed 14 Feb 2019.

[20] Campbell SJ, Savage GB, Gray DJ, Atkinson JA, Soares Magalhaes RJ (2014). Water, Sanitation, and Hygiene (WASH): a critical component for sustainable soil-transmitted helminth and schistosomiasis control. PLoS Negl Trop Dis.

[21] Stothard JR, Khamis AN, Khamis IS, Neo CH, Wei I (2016). Health education and the control of urogenital schistosomiasis: assessing the impact of the Juma na kichocho comic-strip medical booklet in zanzibar. J Biosoc Sci.

[22] Mwakitalu ME, Malecela MN, Mosha FW, Simonsen PE (2014). Urban schistosomiasis and soil transmitted helminthiases in young school children in Dar Es Salaam and Tanga, Tanzania, after a decade of anthelminthic intervention. Acta Trop.

[23] Mwanga JR, Magnussen P, Mugashe CL, Gabone RM, Aagaard-Hansen J (2004). Schistosomiasis-related perceptions, attitudes and treatment-seeking practices in Magu district, Tanzania: public health implications. J Biosoc Sci.

[24] Stothard JR, Mgeni AF, Khamis S, Seto E, Ramsan M, Rollinson D (2002). Urinary schistosomiasis in schoolchildren on Zanzibar Island (Unguja), Tanzania: a parasitological survey supplemented with questionnaires. Trans R Soc Trop Med Hyg.

[25] Koffi AJD, Doumbia M, Fokou G, Keita M, Kone B, Abe NN. Community knowledge, attitudes and practices related to schistosomiasis and associated healthcare-seeking behaviours in northern cote d'Ivoire and southern Mauritania. Infect Dis Poverty. 2018. 10.1186/s40249-018-0453-0. 10.1186/s40249-018-0453-0 PMC6038328 29986766

[26] Chami GF, Kontoleon AA, Bulte E, Fenwick A, Kabatereine NB, Tukahebwa EM (2017). Community-directed mass drug administration is undermined by status seeking in friendship networks and inadequate trust in health advice networks. Soc Sci Med.

[27] Odhiambo GO, Musuva RM, Odiere MR, Mwinzi PN (2016). Experiences and perspectives of community health workers from implementing treatment for schistosomiasis using the community directed intervention strategy in an informal settlement in Kisumu City, western Kenya. BMC Public Health.

[28] Chami GF, Kontoleon AA, Bulte E, Fenwick A, Kabatereine NB, Tukahebwa EM (2016). Profiling nonrecipients of mass drug Administration for Schistosomiasis and Hookworm Infections: a comprehensive analysis of Praziquantel and Albendazole coverage in community-directed treatment in Uganda. Clin Infect Dis.

[29] Tuhebwe D, Bagonza J, Kiracho EE, Yeka A, Elliott AM, Nuwaha F (2015). Uptake of mass drug administration programme for schistosomiasis control in Koome Islands, Central Uganda. PLoS One.

[30] World Health Organization. Schistosomiasis: progress report 2001–2011, strategic plan 2012–2020. World Health Organization. http://wwwwhoint/iris/handle/10665/78074 Accessed 22 Feb 202031.

[31] Inobaya MT, Chau TN, Ng SK, MacDougall C, Olveda RM, Tallo VL (2018). Mass drug administration and the sustainable control of schistosomiasis: community health workers are vital for global elimination efforts. Int J Infect Dis.

[32] Knopp S, Person B, Ame SM, Ali SM, Muhsin J, Juma S (2016). Praziquantel coverage in schools and communities targeted for the elimination of urogenital schistosomiasis in Zanzibar: a cross-sectional survey. Parasit Vectors.

[33] An informal consultation on Schistosomiasis control. Geneva, Switzerland, 30 March −1 April 2011.https://www.who.int/neglected_diseases/resources/9789241505017/en/. Accessed 22 Feb 2020.

[34] WHO. EMRO report of an inter-country meeting on strategies to eliminate schistosomiasis from the Eastern Mediterranean Region, Muscat, Oman, 6–8 November. https://apps.who.int/iris/handle/10665/115981. Accessed 22 Feb 2020.

[35] Katz N, Chaves A, Pellegrino J (1972). A simple device for quantitative stool thick-smear technique in Schistosomiasis mansoni. Rev Inst Med Trop Sao Paulo.

[36] Casacuberta M, Kinunghi S, Vennervald BJ, Olsen A (2016). Evaluation and optimization of the circulating Cathodic antigen (POC-CCA) cassette test for detecting *Schistosoma mansoni* infection by using image analysis in school children in Mwanza region, Tanzania. Parasite Epidemiol Control.

[37] Elmorshedy HBR, El-Ela NE, Eassa SM, Elsakka EE, Barakat R. Can human schistosomiasis mansoni control be sustained in high-risk transmission foci in Egypt? Parasit Vectors. 2015. 10.1186/s13071-13015-10983-13072. 10.1186/s13071-015-0983-2 PMC4502643 26174621

[38] EMH BR, Farghaly A, RS MDM (2014). Human Schistosomiasis in the Middle East and North Africa Region. Neglected Tropical Diseases - Middle East and North Africa Neglected Tropical Diseases.

[39] Corstjens PL, De Dood CJ, Kornelis D, Fat EM, Wilson RA, Kariuki TM (2014). Tools for diagnosis, monitoring and screening of Schistosoma infections utilizing lateral-flow based assays and upconverting phosphor labels. Parasitology.

[40] Haggag AA, Rabiee A, Abd Elaziz KM, Gabrielli AF, Abdel Hay R, Ramzy RMR (2017). Mapping of Schistosoma mansoni in the Nile Delta, Egypt: assessment of the prevalence by the circulating cathodic antigen urine assay. Acta Trop.

[41] World Health Organization. Schistosomiasis and soiltransmitted helminthiases: numbers of people treated in 2017. WHO Weekly Epidemiological Record (2017 and 2018). No 50, 2018, 93, 681–692. .https://www.who.int/wer/en/. Accessed 20 Dec 2019.

[42] Yang K, Sun LP, Liang YS, Wu F, Li W, Zhang JF (2013). Schistosoma japonicum risk in Jiangsu province, People's Republic of China: identification of a spatio-temporal risk pattern along the Yangtze River. Geospat Health.

[43] El Khoby T, Galal N, Fenwick A (1998). The USAID/government of Egypt's Schistosomiasis research project (SRP). Parasitol Today (Personal ed).

[44] El-Khoby T, Galal N, Fenwick A, Barakat R, El-Hawey A, Nooman Z (2000). The epidemiology of schistosomiasis in Egypt: summary findings in nine governorates. Am J Trop Med Hyg.

[45] Elmorshedy H, Tanner M, Bergquist RN, Sharaf S, Barakat R (2016). Prophylactic effect of artemether on human schistosomiasis mansoni among Egyptian children: a randomized controlled trial. Acta Trop.

[46] Peters PA, El Alamy M, Warren KS, Mahmoud AA (1980). Quick Kato smear for field quantification of Schistosoma mansoni eggs. Am J Trop Med Hyg.

[47] Barakat R, Farghaly A, Morshidy HN, el Sayed MK, Masry AG, Husein MH (1995). Patterns of infection, incidence and reinfection with *Schistosoma mansoni* in Nile Delta governorate: Kafr El sheikh. Trop Geogr Med.

[48] Tchuem Tchuente LA, Momo SC, Stothard JR, Rollinson D (2013). Efficacy of praziquantel and reinfection patterns in single and mixed infection foci for intestinal and urogenital schistosomiasis in Cameroon. Acta Trop.

[49] Mitra AK, Mawson AR. Neglected Tropical Diseases: Epidemiology and Global Burden. Trop Med Infect Dis. 2017;2(3). 10.3390/tropicalmed2030036. 10.3390/tropicalmed2030036 PMC6082091 30270893

[50] Tanaka H, Tsuji M (1997). From discovery to eradication of schistosomiasis in Japan: 1847-1996. Int J Parasitol.

[51] Chen J, Xu J, Bergquist R, Li SZ, Zhou XN. “Farewell to the God of Plague”: The Importance of Political Commitment Towards the Elimination of Schistosomiasis. Trop Med Infect Dis. 2018;3(4). 10.3390/tropicalmed3040108. 10.3390/tropicalmed3040108 PMC6306784 30282897

