# Supplementary material for: Elimination of schistosomiasis requires multifactorial diagnostics: evidence from high- and low-prevalence areas in the Nile Delta, Egypt
Source: Infect Dis Poverty. 2020 Apr 3;9:31. doi: 10.1186/s40249-020-00648-9 (PMC7119160; doi:10.1186/s40249-020-00648-9)
Supplement: Supplementary file 1 — Additional file 1. Data collection sheet. [file 40249_2020_648_MOESM1_ESM.pdf]

Schistosomiasis Research Project: EPI 123: Kafr Elsheikh Governorate

The microlevel approach

Data Collection sheet: Round 1

Part I: Personal & sociodemographic data

Respond to the following questions by writing your answer/selecting the answer

1. Village: 1. Ebiana 2. El-Rouse
2. House ID: …….
3. Person ID: …….
4. Gender: 1. Male 2. Female
5. Age: ………
6. Education: 1. Don't read and write 2. Finished primary school
   3. Finished preparatory school 4. Finished secondary school
   4. Bachelor degree or above

7. Occupation: 1. House wife 2. Farmer 3. Fishermen
   4. Students 5. Not working
   6. Others……

Part II: Knowledge about schistosomiasis: please respond to the following questions by choosing the answer that best represent you.

Questions	Responses
8.What is Bilharziasis?	1. A disease<br>2. A Kind of worm<br>3. A kind of water pollution<br>4. A kind of air pollution
9.Which organs are severely affected due to Bilharziasis?	1. All body Organs<br>2. Organs of the genitourinary system<br>3. Organs of the cardio vascular and respiratory<br>4. Organs of the nervous system<br>5. Organs of the GIT system
10.How do people get infected by Bilharziasis?	1. Eating unwashed vegetables<br>2. Skin Contact with canal water<br>3. Drinking canal water<br>3. Contact with diseased persons<br>4. Air pollution<br>5. walking bare footed
11.How does Bilharzia infect people via canal water?	1. Contact with canal and drain waters during farming<br>2. Contact with canal water during fishing<br>3. Contact with canal water during domestic activities<br>4. Swimming in canal water<br>5. Drinking canal water<br>6. Contact with canal water while washing animals

	7. Using canal water for ablution
12.Which sources pollute the canal water?	1. Defecation /urination in water bodies<br>2. Disposal of human excreta into drains<br>3. Washing vegetables in water bodies<br>4. Dumping garbage in water bodies
13.How does human excreta reach the canal?	1. Drainage of latrines into water bodies<br>2. Urination and defecation in water bodies<br>3. using excreta as fertilizers
14.What, and where are snail habitat?	1. In drains only<br>2. In canals only<br>3. In both canals & drains<br>4. on banks of water bodies
15.What is the role of snails in the life cycle?	1. Essential to complete the cycle<br>2. Carry infective stages across water bodies<br>3. not essential to complete the life cycle
16.How can one identify Bilharziasis?	1. Large abdomen<br>2. easy fatigue<br>3. Blood in stools and /or urine<br>4. Urine/stool analysis<br>5. Blood tests
17.What are the types of available treatment	1. Tablets<br>2. Injections<br>3. Suppositories<br>4. Suspension
18.How can one be sure of cure after being treated?	1. Feeling well<br>2. Stool analysis<br>3. Urine analysis<br>4. Blood tests<br>5. Abdominal sonography
19.How can you protect water stream from Bilharzia?	1. Avoid dumping human excreta into water bodies<br>2. avoid dumping garbage into water bodies<br>3. Get rid of snails
20.How can you protect yourself From Bilharzia?	1. Avoid direct contact with water bodies<br>2. By vaccination<br>3. Repeated treatment

Part III: Please respond to the following questions by selecting the response that best describe you:

A (Agree), NC (Not certain), DA (disagree)

Question	A	NC	DA
21. Despite the presence of public tap water, people still use canals.			
22. Despite having clean water indoors, people still use canals			
23. People claim that they are used to handle canal's water, and they had never been infected			
24. Just washing cloths and utensils in canals, doesn't cause bilharziasis			
25. Just irrigating land bare-footed doesn't cause bilharziasis			

26. Swimming in canals once/twice doesn’t cause bilharziasis			
27. Just washing hands, legs or ablution in canals doesn’t cause bilharziasis			
28. Bilharziasis is not a serious infection with potentially grave symptoms			
29. Bilharziasis should not make you seriously worried			
30. Bilharziasis does not seriously affect person’s work capacity			
31. You would never be infected if you had never used canal water			
32. Avoiding urination and defecation in streams will prevent Bilharziasis			
33. Medicine from the Health center can cure Bilharziasis			
34. Repeated urine and stool examination is important			
35. There is no need for treatment because you are going to be infected again			

Part IV: The following questions reflect your opinion about services provided at village health center, select the answer that best describe your opinion.

Question	Response
36. How do you rate the health center as a source of health care?	1. Primary source<br>2. Secondary source<br>3. Not a source<br>4. Never sought health care
37. What is your primary source of health care?	1. Private clinic<br>2. Health center<br>3. MOPH hospitals<br>4. Health insurance unit<br>5. Other sources………….
38. Where did you conduct the last stool analysis?	1. Project<br>2. Health center<br>3. Private physician<br>4. MOPH hospital<br>5. Private laboratory<br>6. School<br>7. Other governmental facilities
39. What is the source of last medical treatment?	1. Project<br>2. Health center<br>3. Private physician<br>4. MOPH hospital<br>5. Pharmacy<br>6. School<br>7. Other governmental facility
40. What is your opinion about the general handling of the patient by the physician at the health center?	1. Good<br>2. Average<br>3. Bad<br>4. Don’t know
41. Do physicians at health center conduct full Patients’ examination?	1. Yes<br>2. Sometimes<br>3. No<br>4. Don’t know

42. How do you find the time spent with patient by the physician?	1. Enough<br>2. Sometimes enough<br>3. Not enough<br>4. Don’t know
43. Do physicians give patients chance to explain condition and symptoms?	1. Yes<br>2. Sometimes<br>3. No<br>4. Don’t know
44. Do physicians provide treatment and condition counselling for patients?	1. Yes<br>2. Sometimes<br>3. No<br>4. Don’t know
45. In general, how do you find the proficiency of the health center?	1. Competent<br>2. Average<br>3. Don’t know
46. In general, how do you find treatment provided in the health center?	1. Good<br>2. Average<br>3. makes me not want to seek treatment at health center<br>4. Don’t know
47. how did you find results of stool and urine analysis at the village health center?	1. Mostly right<br>2. Mostly wrong<br>3. Sometimes wrong<br>4. wrong if extra fees not paid<br>Don’t know
48. Are Bilharziasis treatment’s tablets available at the village health center?	Yes<br>Sometimes<br>No<br>Don’t know
49. How do you find the Cost of diagnosis and treatment of Bilharziasis at the health center?	1. Reasonable<br>2. A lot<br>3. Don’t know
50. On average, what is the travel time to the health center?	1. 1-5 minutes<br>2. 6-10 minutes<br>3. 11-15 minutes<br>4. 16+ minutes
51. What is your opinion about travel time to the health center?	1. Reasonable<br>2. Too much
52. On average, what is the waiting time before seeing the physician at latest visit?	1. 1-15 minutes<br>2. 16-30 minutes<br>3. 31-60 minutes<br>4. 61+<br>5. Doctor didn’t arrive
53. What is your opinion about waiting time at latest visit?	1. Reasonable<br>2. Too much

Part V: The following questions reflect using canal water, select the answer that describe your actual use

Type of activity	Usually	sometimes	Never
54. Farming			
55. Ablution/hand washing			
56. Swimming			
57. Washing cloths			
58. Washing animals			
59. Bringing water from canals			
60. Fishing			

Data Collector

Name Signature

Date
